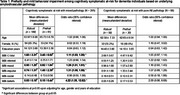# The Association between Mild Behavioral Impairment and Frailty in the setting of Pure Amyloid‐ß and Mixed Pathology in the Southeast Asian BIOCIS Study

**DOI:** 10.1002/alz70857_100635

**Published:** 2025-12-25

**Authors:** Kiirtaara Aravindhan, Yi Jin Leow, Pricilia Tanoto, Gursimar Bhalla, Faith Phemie Hui En Lee, Smriti Ghildiyal, Shan Yao Liew, Kai En Leong, Ashwati Vipin, Bocheng Qiu, Rasyiqah Binte Shaik Mohamed Salim, Gurveen Kaur Sandhu, Jacklyn Leonardo, Fatin Zahra Zailan, Xin Ying Sim, Nagaendran Kandiah

**Affiliations:** ^1^ Lee Kong Chian School of Medicine, Nanyang Technological University, Singapore, Singapore; ^2^ nil, nil, nil, Nicaragua; ^3^ Dementia Research Centre (Singapore), Lee Kong Chian School of Medicine, Nanyang Technological University, Singapore, Singapore; ^4^ Dementia Research Centre (Singapore), Lee Kong Chian School of Medicine, Nanyang Technological University, Singapore 308232, Singapore, Singapore, Singapore; ^5^ Dementia Research Centre (Singapore), Lee Kong Chian School of Medicine, Nanyang Technological University, Singapore 308232, Singapore, Singapore; ^6^ Lee Kong Chian School of Medicine, Nanyang Technological University, Singapore 308232, Singapore, Singapore; ^7^ Neuroscience and Mental Health Programme, Lee Kong Chian School of Medicine, Nanyang Technological University, Singapore, Singapore; ^8^ National Healthcare Group, Singapore, Singapore

## Abstract

**Background:**

Mild behavioral impairment (MBI) and physical frailty are typical syndromes found among older adults and have been attributed to development of cognitive impairment leading to dementia. There is however a paucity of studies observing the association between MBI and frailty in relation to the underlying pathobiology of cognitive impairment including amyloid‐beta (Aß) and/or cerebrovascular pathology. This study therefore examined the associations between MBI and frailty among those who were symptomatic at‐risk for dementia based on their underlying Aß and/or cerebrovascular pathology in a Southeast Asian cohort.

**Method:**

This cross‐sectional study involved 293 community‐dwellers exhibiting subjective cognitive decline or mild cognitive impairment with complete neuropsychological, neuroimaging, behavioural and blood biomarker assessments from the Biomarker and Cognition Study Singapore. The 34‐item Mild Behavioral Impairment Checklist (MBI‐C) and Fried Frailty Phenotype was used to assess MBI and physical frailty respectively. Aß pathology was determined through plasma Aß42/40 ratio ≤0.05, while cerebrovascular pathology was determined through the modified Fazekas rating ≥5. Participants were classified into pure‐Aß or mixed (i.e.vascular and vascular‐ Aß). Independent t‐test and logistic regression were administered to observe differences in non‐frail and prefrail individuals and determine the association between frailty and MBI separately for the two pathologies.

**Result:**

The study population had a mean age of 63.92±8.27, 60.4% females, 31% were prefrail, MBI‐C mean score of 2.59±4.91 and 30% had pure‐Aß pathology. Significant differences (*p* <0.05) in MBI‐C total score and domains of mood, impulse and beliefs were observed between those who were non‐frail and prefrail in the mixed pathology group, the former having higher mean scores than the latter. No similar differences were observed in the pure‐Aß group. Significant associations (*p* <0.05) were present between increased MBI‐C total score (OR:1.11(95%CI:1.02,1.20)) and domains of mood (OR:1.27(95%CI:1.01,1.60)), impulse (OR:1.31(95%CI:1.08, 1.58)) and belief (OR:3.97(95%CI:1.32,11.95)), with likelihood of being prefrail in mixed pathology, upon age, gender and years of education adjustments.

**Conclusion:**

Findings highlight a significant association between MBI and frailty among symptomatic at‐risk individuals having mixed vascular‐amyloid pathology; worse MBI increasing the likelihood of prefrailty. This association highlights a potential behavioural basis for the detection of and/or intervention for cognitive impairment, warranting further investigation.